# Intermittent Preventive Treatment of Malaria in Pregnancy with Sulphadoxine-Pyrimethamine and its Associated Factors in the Atwima Kwanwoma District, Ghana

**DOI:** 10.5334/aogh.3560

**Published:** 2022-04-27

**Authors:** Emmanuel Kumah, Ferguson Duvor, Godfred Otchere, Samuel Egyakwa Ankomah, Adam Fusheini, Collins Kokuro, Akua Kusiwaa Karikari, Joseph Adom

**Affiliations:** 1 Department of Health Administration and Education, Faculty of Science Education, University of Education, Winneba, Ghana; 2 Department of Public Health, Faculty of Health Sciences, Catholic University College of Ghana; 3 Policy, Planning, Monitoring and Evaluation Unit, Komfo Anokye Teaching Hospital, Kumasi, Ghana; 4 Faculty of Humanities, Center for Medicine and Society, University of Freiburg, Germany; 5 Department of Preventive and Social Medicine, Dunedin School of Medicine, University of Otago, Dunedin, New Zealand; 6 Center for Health Literacy and Rural Health Promotion, Accra, Ghana; 7 Department of Medicine, School of Medicine and Dentistry, Kwame Nkrumah University of Science and Technology, Kumasi, Ghana; 8 Directorate of Eye, Ear, Nose and Throat, Komfo Anokye Teaching Hospital, Kumasi, Ghana; 9 Directorate of Oncology, Komfo Anokye Teaching Hospital, Kumasi, Ghana

## Abstract

**Background:**

Intermittent preventive treatment of malaria in pregnancy with Sulphadoxine-Pyrimethamine (IPTp-SP) tablets is one of the recommended interventions to reduce the burden of malaria on both the pregnant woman and the unborn child. The aim of this study was to assess the prevalence of IPTp-SP uptake and its associated factors in the Atwima Kwanwoma District of Ashanti Region, Ghana.

**Methods:**

The study was cross sectional. A structured questionnaire was administered to 394 respondents, comprising pregnant women in their last two months of pregnancy and nursing mothers who delivered within three months prior to the study. Medical records of the respondents were also reviewed. Descriptive statistics such as simple proportions, and averages were computed. Chi-square test and multiple logistic regression analysis were performed to determine factors associated with IPTp-SP uptake.

**Results:**

The average age of the respondents was 28.2 (±5.9) years. Almost all of the respondents (98%) had received SP at the time of the study. Fifty percent received their first dose of SP between 16 and 19 weeks of gestation. The multiple logistic regression analysis showed a statistically significant association between IPTp-SP uptake and educational level, time of first ANC visit, number of ANC visits and receiving education on SP prior to the administration of the drug.

**Conclusion:**

Education on SP use should be intensified at all levels of the health system. Early initiation of ANC is also recommended for optimal uptake of IPTp-SP. More research is needed to understand factors affecting the uptake of SP during pregnancy in the country.

## Background

Malaria remains a major public health concern, especially among pregnant women and children under five years of age [1]. Over 30 million women in Africa become pregnant in malaria endemic areas and are at higher risk of malaria infection caused by Plasmodium falciparum compared to non-pregnant women [1]. In 2018, an estimated 11 million pregnancies were exposed to malaria in sub-Saharan Africa (SSA) [2]. Malaria in pregnancy is often associated with unfavorable outcomes, such as still birth, low birth weight, pre-term delivery, abortion and maternal death [3]. For instance, about 25% of maternal deaths in malaria endemic regions in SSA are attributed to malaria in pregnancy [4].

Ghana is a malaria endemic country, with a population of over 30 million being exposed to the disease all year round [5]. Even though effective interventions have been implemented over the years to protect pregnant women and children, malaria is still a serious public health problem for these vulnerable populations. Available evidence indicates that malaria among pregnant women accounts for about 14% of outpatient services utilization, 11% of admissions and 9% of deaths [6].

Intermittent preventive treatment in pregnancy with sulphadoxine- pyrimethamine ((IPTp-SP) is considered effective in the prevention and control of malaria among pregnant women [7]. The World Health Organization (WHO) recommends that pregnant women in malaria endemic areas should receive a minimum of three doses of sulphadoxine-pyrimethamine (SP) during each pregnancy [1]. IPTp-SP consists of a full therapeutic course of anti-malarial medication administered to pregnant women at routine antenatal care (ANC) visits [7].

Ghana Health Service (GHS) adopted SP as the medicine for IPT in 2003 [8]. The current national policy requires the administration of the drug to all eligible pregnant women during ANC visits, starting from 16 weeks of gestation and repeated monthly until delivery [9]. Although some progress has been made, the country’s ability to achieve the global target of 80% IPTp-SP coverage still remains a challenge. Previous studies on the topic have reported mixed findings. While some have reported high levels of uptake, ranging from 71% [10] to 90.6% [11] in some parts of the country; others have reported below 50% levels of uptake [9,12].

In 2017, Ashanti Region recorded 37.5% coverage for three doses of SP uptake (IPT3). One district contributing to the low performance in the Region is the Atwima Kwanwoma District, with 2017 IPT3 coverage of 33.3% and 36.9% in 2018 [13]. The purpose of this study, therefore, was to examine the prevalence of IPTp-SP uptake and its associated factors in the Atwima Kwanwoma District. Findings from the study could inform and be used by health authorities, such as the Ministry of Health, the National Malaria Control Program, the Ghana Health Service, and other stakeholders to improve IPTp uptake in the study area and other districts in the country with similar challenges.

## Materials and Methods

### Study design and setting

A facility-based descriptive cross-sectional study was conducted among pregnant women and nursing mothers who attended some selected facilities for ANC services in the Atwima Kwanwoma District. The District is one of the 260 Metropolitan, Municipal and District Assemblies (MMDAs) in Ghana, and forms part of the 43 MMDAs in the Ashanti Region. With a population of 143 510, the District is located in the central part of Ashanti Region and has a land size of 251.9 sq.km. The District has 20 health facilities, comprising 11 government owned, seven private and two belonging to the Christian Health Association of Ghana (CHAG) [14].

### Sample size estimation and sampling

A total of 394 pregnant women and nursing mothers were included in the study. This was computed using an estimated IPT3 uptake of 36.9% in the Atwima Kwanwoma District, with a margin of error of 5% and confidence interval of 95%. The calculation was done using the Cochran formula [15]:

N = \frac{{{z^2}pq}}{{{d^2}}}

Where: N = sample size to be determined, z = statistical certainty of 1.96 at a confidence level of 95%, p = proportion of pregnant women who received IPT3 (0.369), q = proportion of those who did not receive IPT3 (1–p = 0.631), and d = margin of error (0.05).

Substituting the above figures:

N = \frac{{{{\left({1.96} \right)}^2}\left({0.369} \right)\left({0.631} \right)}}{{{{\left({0.05} \right)}^2}}} = 358

Non-response rate of 10% was added to give the sample size of 394.

Five health facilities were purposively selected from the list of health facilities providing ANC services in the District. These facilities were Kokoben Health Center, Foase Health Center, Kwanwoma Health Center, Traboum Health Center, and Garry Marvin Memorial Hospital. The five facilities were selected on the basis of their being the major providers of ANC services in the District. All pregnant women in their last two months of pregnancy, attending ANC at the selected facilities or nursing mothers who delivered within three months prior to the study and had attended the selected facilities for ANC services were invited to participate in the study. The 394 respondents were conveniently recruited as follows: 76 from Kokoben Health Center, 74 from Foase Health Center, 70 from Kwanwoma Health Center, 75 from Traboum Health Center, and 99 from Garry Marvin Memorial Hospital.

### Data collection tools and procedure

Data collection techniques employed were review of medical records and questionnaire administration. Medical records of the respondents were reviewed from the clients’ ANC record books. Information extracted included: gestational age at first ANC visit, number of visits made, gestational age at first dose of SP, and doses of SP received. Structured questionnaire was administered via telephone interview to collect data on the respondents’ sociodemographic characteristics (age, marital status, level of education, number of children and occupation), education received on SP, and perceived attitude of ANC staff. The respondents’ telephone numbers were picked from their ANC records at the selected facilities and contacted for the telephone survey. Data collection was done between October and December, 2020 by strictly adhering to the WHO and the government of Ghana’s COVID-19 preventive measures.

Pretesting of the questionnaire was done using 20 ANC attendants and 15 nursing mothers in the Asokwa District which has similar geographical characteristics as Atwima Kwanwoma District. The purpose of the pretesting was to evaluate the effectiveness and consistency of the data collection instrument, as well as knowing the amount of time required to complete a set of questionnaire.

### Data analysis

The collected data was entered into Epi Info 7.0 and analyzed using Statistical Package for Social Sciences (SPSS) software version 20 (IBM© Corporation, Armonk, NY, USA). IPTp-SP uptake, the outcome variable, was categorized into sub-optimal (≤ 2 doses), and optimal (≥ 3 doses). Descriptive statistics were used to present the demographic data of the respondents. Chi-square test was conducted to test the level of significance and association between IPTp-SP uptake and each independent variable. Multiple logistic regression analysis was then used to identify significant predictors of IPTp-SP uptake, including all of the explanatory variables that were found to be statistically significant after the Chi-square test. For all statistical test in this study, a level of statistical significance was set at a p-value of 0.05.

### Ethical consideration

The study protocol was approved by the Ethics Review Committee of the Ghana Health Service Research and Development Division, Accra (Ref: GHS-ERC-027/02/20) and the Graduate and Research Department of Catholic University College of Ghana. The District Health Director in Atwima Kwanwoma, as well as health facility heads were formally written to for permission to conduct the study. All information captured was treated confidentially.

## Results

### Demographic characteristics of the respondents

Of the 394 women who participated in the study, 57% were pregnant women, while the remaining 43% were nursing mothers. The average age of the respondents was 28.2 (±5.9) years, with the majority being married (79.2%), having completed senior high school (60.9%), employed (84%), and being Christian (74%). ***Table 1*** shows details of the background characteristics of the study respondents.

**Table 1:** Demographic characteristics of the study respondents (n = 394).


	FREQUENCY	PERCENT

**Age in years: mean ± SD**	28.19 ± 6.40	

≤19	37	9.39

20–24	76	19.29

25–29	104	26.4

30–34	98	24.87

≥35	79	20.05

**Marital status**		

Married	312	79.19

Divorced	12	3.05

Single	50	12.69

Cohabitation	20	5.08

**No. of children**		

No child	44	11.17

1–2	210	53.3

3–4	101	25.63

5–6	39	9.90

**Level of education**		

No formal education	19	4.82

Primary	69	17.51

Junior high	99	25.13

Senior high	141	35.79

Tertiary	66	16.75

**Occupation**		

Employed	332	84.26

Unemployed	62	15.74


SD: Standard Deviation.

### ANC services utilization and uptake of IPTp-SP

The majority of the women (55%) made their first ANC visit during the first trimester, with about 38.6% making the first visit during the second trimester. On average, the respondents visited ANC for the first time after 16.4 weeks of gestation. Almost all of the respondents (98%) had received SP at the time of the study, with about 46% receiving at least three doses. Also, 50% of them received their first dose of SP between 16 and 19 weeks of gestation. Education on SP was given to 63% of the women prior to the administration of the medication, with 37% indicating they received no education. In terms of staff attitude, 61.4% described the attitude of staff at the ANC clinics as positive (good or excellent) (***Table 2***).

**Table 2:** ANC services utilization and IPTp-SP uptake.


VARIABLE	NO.	%

**Gestational age at first ANC visit:**		

First trimester	217	55.08

Second trimester	152	38.58

Third trimester	25	6.35

**Number of ANC visits:**		

1–4	82	20.68

5–7	165	41.92

≥8	147	37.40

**Gestational age at first dose of SP:**		

16–19 weeks	193	48.98

20–23 weeks	152	38.58

≥24 weeks	49	12.44

**Doses of IPTp-SP received:**		

≤2 doses	213	54.06

≥3 doses	181	45.94

**Prior education on SP:**		

Yes	249	63.20

No	145	36.80

**Perceived attitude of ANC staff:**		

Good	242	61.42

Poor	152	38.58


### Factors associated with IPTp-SP uptake

Our bivariate analysis revealed that age, marital status, number of children one has, and perceived attitude of ANC staff had no significant association with IPTp-SP uptake. In all, six variables, namely: level of education, occupation, gestational age at first ANC visit, number of ANC visits, gestational age at first dose of SP and receiving education on SP prior to the administration of the medication, were significantly associated with the uptake of IPTp-SP (***Table 3***).

**Table 3:** Bivariate analysis of factors associated with IPTp-SP uptake (n = 394).


VARIABLE	≤2 DOSES OF SP	≥3 DOSES OF SP	X^2^	P VALUE

**Age:**				

≤19	18 (48.6%)	19 (51.4%)	0.85	0.393

20–24	42 (55.3%)	34 (44.7%)		

25–29	57 (54.8%)	47 (45.2%)		

30–34	52 (53.1%)	46 (46.9%)		

≥35	44 (55.7%)	35 (44.3%)		

**Marital status:**				

Married	170 (54.5%)	142 (45.5%)	0.68	0.912

Divorced	6 (50.0%)	6 (50.0%)		

Single	26 (52.0%)	24 (48.0%)		

Cohabitation	11 (55.0%)	9 (45.0%)		

**No. of children:**				

No child	22 (50.0%)	22 (50.0%)	0.27	0.961

1–2	116 (55.0%)	94 (45.5)		

3–4	55 (54.5%)	46 (45.6%)		

5–6	20 (51.3)	19 (48.7%)		

**Level of education:**				

No formal education	11 (57.9%)	8 (42,1%)	21.47	0.012

Primary	36 (52.2%)	33 (47.8%)		

Junior high	55 (55.6%)	44 (44.4%)		

Senior high	75 (53.2%)	66 (46.8%)		

Tertiary	36 (54.5%)	30 (44.5)		

**Occupation:**				

Employed	176 (53.0%)	156(47%)	5.41	0.048

Unemployed	37 (59.7%)	25 (40.3%)		

**Gestational age at first ANC visit:**				

First trimester	115 (53.0%)	102 (47.0%)	31.20	0.001

Second trimester	84 (55.3%)	68 (44.7%)		

Third trimester	14 (56.0%)	11 (44.0%)		

**Number of ANC visits:**				

1–4	44 (53.7%)	38 (46.3%)	25.28	0.002

5–7	89 (53.9%)	76 (46.1%)		

≥8	80 (54.4%)	67 (45.6%)		

**Gestational age at first dose of SP:**				

16–19 weeks	104 (53.9%)	89 (46.1%)	19.65	0.021

20–23 weeks	82 (54.0%)	70 (46.0%)		

≥24 weeks	27 (55.1%)	22 (44.9%)		

**Prior education on SP:**				

Yes	135 (54.2%)	114 (45.8%)	9.74	0.032

No	78 (53.8%)	67 (46.2%)		

**Perceived attitude of ANC staff:**				

Good	131 (54.1%)	111 (45.9%)	1.98	0.557

Poor	82 (53.9%)	70 (46.1%)		


Results of the logistic regression analysis (***Table 4***) revealed that factors significantly associated with the uptake of ≥3 doses of SP for IPTp were: educational level, time of first ANC visit, number of ANC visits and receiving prior education on SP. We observed that pregnant women with higher levels of education were two times more likely (AOR = 2.3, 95% CI: 0.95–4.87), compared to those with lower levels of education, to receive ≥3 doses of SP, after adjusting for the relationships of the other independent variables. Also, women who initiated ANC in the second and third trimesters had lower odds (AOR = 0.72, CI: 0.43–1.02; and AOR = 0.58, CI: 0.23–1.25 respectively) of receiving ≥3 doses of SP compared to those who initiated ANC in the first trimester. Furthermore, women who made more than five ANC visits were 5.6 times more likely (AOR = 5.62, CI: 2,33–7.55), than those who made five or less visits, to complete at least three doses of SP. Finally, respondents who indicated that they were given education prior to the administration of SP had higher odds (AOR = 1.96, CI: 0.85–3.12) of completing three or more doses compared to those who had no education prior to the administration of the medication.

**Table 4:** Multiple logistic regression analysis of factors associated with ≥3 doses of SP.


VARIABLE	COR (95%CI)	AOR (95%CI)

**Level of education:**		

Non/Primary/Junior High	Ref	Ref

Senior high/Tertiary	2.66 (1.18–5.99)	2.3 (0.95–4.87)*

**Occupation:**		

Unemployed	Ref	Ref

Employed	1.83 (0.6–2.01)	1.52 (0.53–1.92)

**Time of first ANC visit:**		

First trimester	Ref	Ref

Second trimester	0.84 (0.58–1.39)	0.72 (0.43–1.02)*

Third trimester	0.62 (0.34–1.33)	0.58 (0.23–1.25)*

**Number of ANC visits:**		

≤5	Ref	Ref

>5	6.86 (2.75–8.96)	5.62 (2.33–7.55)*

**Gestational age at first ANC visit:**		

≤20	Ref	Ref

>20	0.74 (0.28–2.46)	1.23 (0.58–2.84)

**Prior education on SP:**		

No	Ref	

Yes	2.09 (1.31–3.45)	1.96 (0.85–3.12)*


* Statistically significant (p < 0.05). COR = crude odds ratio, AOR = adjusted odds ratio, CI = confidence interval.

## Discussion

Uptake of at least three doses of SP is known to be effective in reducing malaria in pregnancy, maternal anemia, low birth weight and neonatal mortality [16]. We conducted this study to assess the level of uptake of IPTp-SP and its associated factors in the Atwima Kwanwoma District of the Ashanti Region, Ghana. Of the 394 women who participated in the study, 46% received ≥3 doses of IPTp-SP as recommended by WHO. Educational level, time of first ANC visit, number of ANC visits and receiving education on SP prior to its administration were the factors found to be significantly associated with IPTp-SP uptake.

The 46% of pregnant women receiving ≥3 doses of IPTp-SP found in this study is above what was reported (36.9%) in the District in 2018 by the National Malaria Control Program. However, the present finding and those from previous studies conducted in other parts of the country have reported different levels of IPTp-SP uptake. For instance, Amoako and Anto [11] reported 90.6% level of IPTp-SP uptake in the Cape Coast Metropolis, Ibrahim et al [17]. reported an uptake of 71% in Sunyani in the Bono Region, while Addai-Mensah et al [9]. found the level of IPTp-SP uptake to be 32% at the University Hospital, Kumasi. This observation calls for nationwide studies, employing larger samples, to determine the current level of IPTp-SP uptake in the country. One of the few attempts to fill this gap is a 2019 secondary data analysis of the 2016 Ghana Malaria Indicator Survey (GMIS) by Darteh and colleagues. The authors reported that the overall proportion of women in Ghana with ≥3 doses of IPTp-SP uptake during pregnancy was 63% [18]. However, the study was not published in an academic peer-reviewed journal, and thus its methodological quality could not be vigorously assessed.

We observed that attaining higher level of education significantly influence the uptake of ≥3 doses of SP. This is consistent with findings from earlier studies [9,17,19,20,21]. The explanation has been that women with secondary and tertiary levels of education are able to read books and newspapers, as well as listening to radio and watching TV to understand the effects of malaria in pregnancy [19,20]. These women better understand the effects of malaria on themselves and their future newborns [17]. They also appreciate the benefits of IPTp-SP and the adverse effects of placental malaria regarding low birth weight and other birth outcomes [21]. Thus, such women are motivated to take the recommended doses of SP to avoid the adverse effects of malaria in pregnancy.

Early initiation and high utilization of ANC services are known to predict the uptake of IPTp-SP [22]. We observed in this study that women who initiated ANC in the first trimesters had higher odds of receiving ≥3 doses of SP compared to those who initiated ANC in the second and third trimesters. Also, women who made more than five ANC visits were 5.6 times more likely, than those who made five or less visits, to complete at least three doses of SP. These findings could be explained by the fact that early initiation of ANC might result in one’s ability to make the recommended minimum of four visits and thus able to receive more doses of SP as suggested by other studies [23,24].

The present study supports a previous study [11] suggesting that women with sufficient knowledge about SP prior to its administration tend to take in more doses of the medication. Knowledge of the use of SP, including when it is supposed to be taken, interval between each dose and any possible side effects could improve women’s decision-making on the uptake of the medication during pregnancy [11].

There are some limitations to the findings of this study. The limited sample size and the restriction of the study to only one district within the Ashanti Region of Ghana limit the generalizability of the findings to the entire country. Another limitation concerns the recruitment of some of the women (57%) into the study at a time they had not delivered. Some could have taken in more doses of SP by delivery and, thus, increased the level of uptake. These limitations notwithstanding, the data triangulation (survey and review of medical records) and the rigorous analytical approach employed ensured the findings were not compromised.

## Conclusions

This study has provided valuable information to inform policy decisions on planning and implementing programs to improve the uptake of IPTp-SP in the Atwima Kwanwoma District, the Ashanti Region and the country as a whole. The study has highlighted the importance of early registration and high utilization of ANC services to the uptake of IPTp-SP among pregnant women. The study has also shown the usefulness of educating pregnant women on SP prior to the administration of the medication. We recommend that women in their fertility age should be encouraged to seek early ANC services when pregnant and have regular visits to increase their uptake of SP for better pregnancy outcomes. Also, education on IPTp should be intensified at all levels of the health system to help pregnant women have adequate knowledge of SP prior to the administration of the medication. In the long-term, formal education of the girl child should be encouraged to improve the educational levels of women in the District so they could read well and appreciate the full benefits of IPTp-SP.

## Data Accessibility Statement

All relevant data are within the paper.
